# Perceived discrimination and its association with self-rated health, chronic pain, mental health, and utilization of health services among Syrian refugees in Norway: a cross-sectional study

**DOI:** 10.3389/fpubh.2024.1264230

**Published:** 2024-02-09

**Authors:** Omid Dadras, Esperanza Diaz

**Affiliations:** ^1^Department of Global Public Health and Primary Care, University of Bergen, Bergen, Norway; ^2^Department of Addiction Medicine, Haukeland University Hospital, Bergen, Norway

**Keywords:** Syrian refugees, perceived discrimination, self-rated health, pain symptoms, mental health, healthcare utilization

## Abstract

**Background:**

There is a scarcity of research on discriminatory experiences and their association with health outcomes among Syrian Refugees in Norway. Thus, this study aims to examine the relationship between perceived discrimination, self-rated health (SRH), chronic pain, poor mental health, and healthcare utilization among Syrian refugees resettled in Norway.

**Methods:**

Cross-sectional data from the Integration for Health project were analyzed, including 154 Syrian refugees who resettled in Norway in 2018–19. Perceived discrimination, SRH, chronic pain, psychological distress, post-traumatic stress symptoms, and healthcare visits were assessed. Statistical analyses, including Poisson regression and multinomial logistic regression, were conducted. The significant statistical level was set at 0.05.

**Results:**

Approximately 30% of participants reported experiencing discrimination, with no significant associations between sociodemographic factors and perceived discrimination. Perceived discrimination was significantly associated with psychological distress (adjusted PR: 2.07, 95%CI: 1.21–3.55), post-traumatic stress symptoms (adjusted PR: 11.54, 95%CI: 1.25–106.16), and 4 or more psychologist visits (adjusted OR: 12.60, 95%CI: 1.72–92.16). However, no significant associations were found between perceived discrimination and SRH; pain symptoms, or general healthcare utilization.

**Conclusion:**

Experienced discrimination is highly prevalent and seems to be associated with mental health outcomes, but not clearly with SRH, pain, or general healthcare visits among Syrian refugees living in Norway. Efforts should focus on reducing discrimination, promoting social inclusion, and improving access to mental health services for refugees. Public awareness campaigns, anti-discrimination policies, and cultural training for healthcare professionals are recommended to address these issues and improve the well-being of Syrian refugees in Norway.

## Introduction

The appropriate integration of refugees in their new home countries is a global humanitarian concern, with millions of individuals forced to leave their home countries due to conflicts and persecution. Among these displaced populations, Syrian refugees constitute one of the largest groups worldwide (1). The Syrian refugee crisis has led to the displacement of millions of individuals who have fled their homes due to violence, persecution, and war. Since the start of the conflict in 2011, more than six million Syrians have been internally displaced, and around five million have sought asylum in other countries or have been placed in refugee camps worldwide (2). As of 2021, UNHCR reported that more than a million Syrian asylum-seekers and refugees ended up in the European Union (EU), with Germany and Sweden being the main host countries (3). In Norway, the Syrian immigrant population increased substantially between 2011 and 2018 (4). Since September 2015, more than half of the asylum seekers to Norway have been from Syria, with a total of 30,110 asylum seekers arriving in Norway in that year (5). The latest data from 2023 indicates 42,397 Syrian refugees are currently living in Norway (6).

Discrimination is defined as the unequal treatment of individuals or groups based on their race, ethnicity, nationality, religion, or immigration status. It can be experienced in various forms, including verbal abuse, physical violence, and social exclusion (7). Although Norway is known for its commitment to human rights and social justice, Syrian refugees living in Norway may still face discrimination, which can have negative impacts on their health and access to healthcare (8, 9). In examining the report from Norway in 2018, the United Nations Committee on the Elimination of Racial Discrimination highlighted concerns about the lack of equal treatment in recruitment processes and the disproportionately high unemployment rate among immigrants in Norway (10). Additionally, a survey in 2020 by the Norwegian Institute for Social Research revealed that the number of Norwegians who believe discrimination occurs “to a large degree” has tripled in the last 6 years, indicating an increasing acknowledgment of discrimination against immigrants in Norway (11). In a study on perceived discrimination among migrants in Norway in 2016, approximately 27% of participants reported perceived discrimination, ranging from 12.8% among Sri Lankans to 38.7% among Iranian Immigrants (12). However, the absence of Syrian refugees in the sample necessitates further investigation to delineate their specific experiences of discrimination.

Discrimination, both perceived and experienced, is recognized as a significant stressor that can negatively impact the mental and physical health of immigrants (12, 13). Discrimination based on race, ethnicity, nationality, religion, or immigration status is consistently associated with poor mental health outcomes in refugees and asylum seekers in general (14). Regular discrimination in daily life has been linked to individuals’ overall poor self-assessment of health and a higher likelihood of experiencing one or more chronic diseases (15). A study in 2016 among migrant groups in Norway observed poorer mental health among migrant groups with discriminatory experiences, however, the Syrian refugees were not represented in this study (12). There might be differences among refugee groups that have to be better understood, as failing to recognize or acknowledge discrimination can have negative health consequences for some individuals from specific groups (16). Despite Syrians being the fourth-largest migrant group in Norway (6), there remains limited research addressing their specific needs. Studies have been conducted on the health-related quality of life and mental distress of Syrian refugee youth resettled in Norway (7), the use of healthcare services among Syrian refugees migrating to Norway (8), and mental health in adult refugees from Syria resettled in Norway (9). However, there is no specific information on the unique discriminatory experiences and consequent psychological outcomes among Syrian refugees in Norway.

Chronic pain is a common health problem that can significantly affect an individual’s function and quality of life. Different forms of pain have been linked to stress through the concept of allostatic load, which is defined as the cumulative wear and tear on the body due to adapting to adverse physical or psychosocial situations such as those caused by perceived discrimination in migrants (17, 18). Recent evidence has indicated that immigrants and refugees may face unique challenges in managing pain conditions, which could be exacerbated by the experience of discrimination (19–21); however, no research has specifically focused on the association between perceived discrimination and chronic pain among Syrian migrants in Norway. Understanding the association between perceived discrimination and these specific pain conditions is crucial for developing targeted interventions and improving the health outcomes of this migrant group.

Discrimination can also limit refugees’ access to regular healthcare, exacerbating physical and mental health problems and creating barriers to recovery and well-being (22, 23). Experiences with discrimination can lead to decreased trust, avoidance of healthcare services, and disparities in health outcomes and could be associated with delays in seeking medical care and poor adherence to medical care recommendations (24, 25). Additionally, refugees and asylum seekers face several barriers to accessing healthcare (26) and evidence shows unmet healthcare needs among specific groups (27), especially when it comes to mental and dental health in the EU member states (28, 29). However, there is limited knowledge of the unique experiences of different migrant groups, particularly Syrian refugees, in accessing healthcare services and the extent to which discrimination affects their health outcomes in Norway (30). Lack of appropriate health care can in turn lead to adverse outcomes like increased diabetes comorbidities (31), and low adherence among hypertensive patients (32). In the latest report on the National strategy to equalize social health differences by the Norwegian Ministry of Health and Care Services, discrimination in access to healthcare among migrants and minorities has been acknowledged repeatedly (33); however, the report failed to specifically address this issue which makes it necessary to explore more the underlying causes and produce clear evidence for the Norwegian context. Additionally, we need granularity of evidence for specific migrant groups to account for both differences in religious and cultural backgrounds of refugees this case Syrian refugees well as sociopolitical contexts of host country, in this case Norway.

Against this background, the purpose of this paper is to examine the relationship of perceived discrimination with self-rated health, pain conditions, poor mental health, and utilization of health services among Syrian refugees resettled in Norway in 2018–19 using the latest data from the Integration for Health project (I4H). In this study, we explored the discrimination experiences of Syrian refugees in Norway and provided valuable knowledge for the association of such experiences with the health challenges they face and their utilization of health services in Norway.

## Methods

### Study design, setting, and population

We used cross-sectional data from the I4H project, which followed up on the Changing Health and Health Care Needs Along the Syrian Refugees’ Trajectories to Norway (CHART) project (34, 35). In the first wave of CHART, all adults (16 + years) Syrian refugees in Lebanon, who were deemed to move to Norway according to the UNHCR quota at two specific missions facilitated by the International Organizartion of Migrants (IOM), were invited, recruited, and interviewed (2016–17) (*N* = 506, 98% of invited). The same cohort was followed up in wave 2 of the CHART project a year after settlement in Norway (2017–18) of which 464 could be tracked and invited for an interview and 350 completed the interview. In I4H, the same cohort (*n* = 464) was approached and invited for an interview approximately 4 years after living in Norway (2021–22) and 154 completed the interview. The interview was conducted on the phone by bilingual interviewers (Arabic, Norwegian) using a researcher-developed questionnaire on the SurveyXact platform. The main reason for attrition (154/464) in the I4H project was the inability to reach out to the participants due to changes in their contact information or not responding after three phone contact attempts. In addition, two deaths and one deaf participant were excluded. The interviews were conducted from September 2022 to February 2023. The questionnaire was developed by the Int4Health team including bilingual members (Arabic, Norwegian), and was based on the previous CHART questionnaires. The questionnaire collected data on the participants’ scoiodemograhy, physical health, mental health, perceived discrimination, and integration using validated questions from relevant standard scales. The questionnaire was translated into the Arabic language by a professional entity and the accuracy and comprehensibility of the questionnaires were assessed through a pilot study recruiting 11 Syrian refugees during which attempts were made to replicate the real interview scenario by two bilingual interviewers (Arabic, Norwegian) supervised by a bilingual advisor (Arabic, Norwegian). The questionnaire was revised minorly after the pilot study to enhance the comprehensibility of the questionnaire.

### Study variables

#### Independent variables

Sociodemographic variables included age, sex, marital status, education level, annual income, having children, and number of children. Perceived discrimination was assessed by asking about the frequency of experiencing discrimination by authorities, at work/school, by nationality, and by race using a 5-point Likert scale (never, seldom, sometimes, often, very often), using four validated questions from the refugee post-migration stress scale (RPMS) which is proved to be a valid instrument among Syrian refugees (36). A composite variable for perceived discrimination was further created by combining the four types of discrimination with two alternative responses (0 “never/seldom,” 1 “sometimes/often/very often”).

#### Dependent variables

Self-rated health (SRH) was measured by asking “How do you consider your health at the moment?” on a 5-point Likert scale (1 “very bad,” 2 “bad,” 3 “fair,” 4 “good,” and 5 “very good”), a validated question from the European Social Survey 2010 (37), this variable further recoded as 1″ poor,” 2″ fair,” and 3 “good” (38). Chronic pain (lasted >6 months) was assessed by asking “Do you have physical pain now that has lasted more than 6 months?,” a single, validated question from the Trøndelag Health Study (HUNT) study (39). Psychological distress was assessed by the Hopkins Symptom Checklist (HSCL-10), which required participants to reflect on their experiences over the past week and quantify the severity of various anxiety and depression symptoms using a 4-point Likert scale. A mean HSCL-10 score of 1.85 (range 1–4) was used as a cut-off indicating clinically relevant psychological distress (40). Post-traumatic stress symptoms (scores>2.5 on the HTQ scale) were assessed by the Harvard Trauma Questionnaire (HTQ) on a 4-point Likert scale with a mean HTQ score of 2.5 (range 1–4) as a threshold, indicating the possibility of clinically significant symptoms relevant to post-traumatic stress disorder (PTSD) (41). GP/specialist/psychologist visits were measured by asking “During the last 12 months, have you visited any of the following?” with three alternative responses (No, 1–3 times, 4 or more times), a question from the HUNT study (39).

### Statistical analysis

Descriptive statistics were used to present the distribution of sample characteristics and prevalence of perceived discrimination by type, as well as the prevalence of outcomes of interest including SRH, chronic pain, psychological distress, post-traumatic stress symptoms, and GP/specialist/psychologist visits in the past 12 months. Principal Component Analysis (PCA) was employed to facilitate the construction of a composite variable for perceived discrimination, intended for subsequent utilization in multivariate analysis. Combining the four questions related to discrimination was instigated by the low number of positive responses for each question which hindered reliable and powerful individual multivariate analyses. This approach was supported by the results produced in PCA analysis in which we realized that all four questions reflect the same construct (perceived discrimination). This was evidenced by the emergence of a single factor with an eigenvalue of more than 1 with all questions loaded on this factor. Thus, we used four questions related to discrimination to create a composite variable of perceived discrimination with two alternative responses (never/rarely, sometimes/often). The chi-square test was used to examine the relationship between perceived discrimination and sociodemographic factors. Poisson regression with robust variance error (vce) for binary outcomes (42) (chronic pain, psychological distress, and PTSD) and multinomial logistic regression for multinomial outcomes (Self-rated health, GP/specialist visits) were used to assess the relationship between perceived discrimination and each outcome of interest in separate models before and after adjustment for potential confounders based on the previous literature, namely, age, sex, education, and income (43–45). To account for non-response and attrition biases, we calculated outcome-specific sampling weights based on participants’ characteristics from the baseline survey in Lebanon (CHART wave 1) that predicted the attrition in the H4I project (age, sex) and applied them in bivariate and multivariate analyses. Results were reported as frequency (%), mean ± standard deviation (year), prevalence ratio (PR), and odds ratio (OR), with a value of p set at 0.05. All the analyses were carried out in STATA version 17.

## Results

### Sociodemographic characteristics of participants

Out of 464 participants at the baseline of the CHART study in Lebanon, 154 (33.19%) with a median age of 41 (IQR = 33.5–46) consented and completed the interview 3–4 years after their resettlement in Norway. In the sample, 54% were female,75% were married and 62% had lower secondary or less education (Table 1). Of 154 respondents, 130 had children with a median number of 4 (IQR = 3–5).

**Table 1:** Distribution of respondents’ sociodemographic characteristics (*n* = 154), by perceived discrimination.

		Perceived discrimination (sometimes/often)	
	*N* (%)	*N* (%)	Value of *p*^a^
Sex			
Female	83 (53.9)	20 (24.1)	
Male	71 (46.1)	25 (35.2)	0.131
Age in years (median, IQR)	41 (33–46)	38 (23–47)	–
Age group			
20–35	45 (30.4)	16 (35.6)	
36–45	61 (41.2)	15 (24.6)	
46–73	42 (28.4)	12 (28.6)	0.468
Education level			
Lower secondary education/less	91 (62.3)	24 (26.4)	
Upper secondary education	34 (23.3)	14 (41.2)	
Higher education	21 (14.4)	6 (28.6)	0.272
Education in years (median, IQR)	8 (6–11)	9 (6–11)	–
Income			
Under 250,000 NOK (low)	62 (40.8)	25 (40.3)	
250,000–449,999 NOK (moderate)	78 (51.3)	17 (21.8)	
Above 450,000 (high)	12 (7.9)	3 (25.0)	0.054
Marital status			
Single	13 (8.4)	5 (38.5)	
Married	116 (75.3)	31 (26.7)	
Divorced/separated/widow	20 (13.0)	8 (40.0)	
Others	5 (3.3)	1 (20.0)	0.529
Had children			
Yes	130 (84.4)	38 (29.2)	
No	24 (15.6)	7 (29.2)	0.995
Children number (median, IQR)^b^	4 (3–5)	3 (2–5)	–

^a^Value of *p* for the chi-square test; only for categorical variables. ^b^Among those who had children.

### Prevalence of perceived discrimination and associated sociodemographic factors

**Figure 1:**
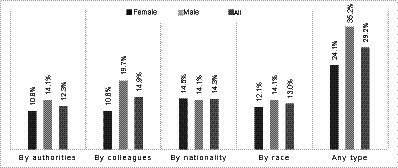
Prevalence of different types of perceived discrimination (sometimes/often), by sex.

An estimated 12.3% (7.6–18.6), 14.9% (9.7–21.6), 14.3% (9.2–20.8), 13.0% (8.1–19.3), and 29.2% (22.2–38.1) of the participants sometimes or often experienced discrimination by authorities, by colleagues at wo1rk/school, by nationality, by race, or any type of discrimination, respectively (Figure 1). Although the prevalence of the perceived discrimination appeared to be higher among males (35.2%), the youngest (35.6%), those with upper secondary education (41.2%), those with a lowest annual income (40.3%), and in the divorced/separated/widow group (40.0%); no statistically significant association was observed between these sociodemographic factors and perceived discrimination (Table 1).

### Relationship between perceived discrimination and outcome variables

Almost half of respondents reported good SRH and approximately a fifth rated their health as poor. As shown in Table 2, the weighted prevalence of the different symptoms varied from 50% for chronic pain to 5% reporting PTSD symptoms. An estimated 87.2% of participants had at least one GP visit in the past 12 months, while 28.7 and 8.6% of participants visited specialists and psychologists at least once during the same period. In both adjusted and unadjusted models, perceived discrimination was only associated with psychological distress (adjusted PR = 2.07, 95%CI: 1.21–3.55), PTSD (adjusted PR = 11.54, 95%CI: 1.25–106.16), and visiting psychologist 4 times or more (adjusted OR = 12.60, 95%CI: 1.72–92.16).

**Table 2:** Prevalence of physical symptoms, mental health complaints, and utilization of healthcare and their association with perceived discrimination (*n* = 154).

		Perceived discrimination (sometimes/often)	
	*N* (weighted%)	PR/OR (95%CI)^a^	Adjusted PR/OR (95%CI)^b^
Self-rated health			
Poor	33 (20.7)	1.12 (0.44–2.83)	1.47 (0.39–5.53)
Fair	41 (27.4)	1.62 (0.70–3.73)	2.26 (0.10–5.99)
Good	80 (51.9)	Reference	Reference
Chronic pain	79 (50.2)	0.87 (0.59–1.26)	0.91 (0.65–1.28)
Psychological distress	43 (29.0)	1.99 (1.21–3.26) *	2.07 (1.21–3.55) *
PTSD symptoms	7 (5.0)	15.09 (1.86–122.60) *	11.54 (1.25–106.16) *
GP visits			
No	20 (12.8)	Reference	Reference
1–3 times	50 (32.0)	0.43 (0.12–1.48)	0.52 (0.12–2.10)
4 times or more	84 (55.2)	1.24 (0.42–3.64)	1.90 (0.49–7.41)
Specialist visit			
No	109 (71.3)	Reference	Reference
1–3 times	35 (22.1)	1.90 (0.83–4.37)	1.96 (0.83–4.67)
4 times or more	10 (6.6)	1.21 (0.29–5.04)	0.89 (0.18–4.48)
Psychologist			
No	140 (91.4)	Reference	Reference
1–3 times	8 (5.2)	1.56 (0.35–6.93)	1.68 (0.31–9.11)
4 times or more	5 (3.4)	12.20 (1.31–113.90) *	12.60 (1.72–92.16) *

^a^PR, prevalence ratio for the binary outcome including chronic pain, psychological distress, and PTSD symptoms. OR (odds ratio) for multinomial outcomes including GP, specialist, and psychologist visits. ^b^Adjusted for age, sex, education, and income as confounding factors. *p-value < 0.05.

## Discussion

To the best of our knowledge, this is one of the first studies exploring the prevalence of perceived discrimination and its association with physical and mental well-being, and utilization of health services among Syrian refugees in Norway. The findings indicated relatively high perceived discrimination among the study population and almost 30% of respondents reported sometimes or often experienced one type of discrimination. Despite the small sample size in our study, the prevalence of perceived discrimination among Syrian refugees in this study approximates the one from a recent survey in Norway. This survey involved 4,294 migrants aged 16–66 years from 12 countries including Poland, Turkey, Bosnia-Herzegovina, Kosovo, Eritrea, Somalia, Afghanistan, Sri-Lanka, Iraq, Iran, Pakistan, and Vietnam, the reported prevalence of perceived discrimination was 27% (12). Although Syrian refugees were not represented in this survey, our finding suggested similar rate of perceived discrimination as other migrant groups in Norway. However, it was slightly lower than a German study in which the prevalence of perceived discrimination was 36% among a smaller sample of 116 Syrian refugees (46). Previous studies consistently reported a high prevalence of perceived discrimination in other migrant groups from different countries of origin and destination (45, 47–49). However, the discrepancies in reported prevalence could be due to several reasons such as the sample size, and methodological differences, and also highlight the influence of cultural and sociopolitical differences in the discriminatory experiences of various migrant groups.

In our study, the prevalence of perceived discrimination appeared to be higher among specific groups such as men, individuals who were divorced/separated/widowed, those with upper secondary education, and individuals with low annual income; however, these associations were not statistically significant. This could be due to the sample size which was not large enough to detect subtle differences or that there was considerable variability within the demographic groups examined, which could have masked significant associations. With regard to the sociodemographic background of refugees and its relation to perceived discrimination, the results from previous studies are also inconsistent (12, 46, 48). For instance, a study in Spain found higher perceived discrimination among migrant men from Ecuador, Morocco, Romania, and Colombia as compared to women (50), while another study in Canada found a higher prevalence among a sample of migrant women from Ethiopia, Korea, Iran, Vietnam, and Ireland (51). Gender-responsive data on migration has been suggested as a potential solution to promote greater equality and offer insights into the specific vulnerabilities and experiences of women and men in migration processes (52). There is also no clear consensus on whether wealth and education could influence the experiences of discrimination among migrants and refugees. For example, research in Sweden found that many asylum seekers, including those with higher education, experience discrimination in the labor market (53), while another study from the United States did not find any association between these two and perceived discrimination (54) which is in line with our findings. These discrepancies, however, could be attributed to the specific context and characteristics of the study population, sample size, differences in instruments and scales, and discrepancies in methodological approaches across studies that can influence the observed relationships between perceived discrimination and demographic factors (13).

The present study found no relationship between perceived discrimination and SRH and chronic pain. In contrast, previous research has reported significant associations between perceived discrimination and various physical symptoms such as headaches, generalized musculoskeletal pain, and low back pain (13, 55). It is important to consider the complex and multifaceted nature of physical symptoms and the potential influence of various mediators and moderators (13). Perceived discrimination may interact with other psychosocial factors, such as stress, coping mechanisms, social support, and cultural factors, which can collectively contribute to the development and expression of physical symptoms (13, 56, 57). Some cultures may view physical symptoms as a sign of weakness or a lack of willpower, while others may view them as a natural part of life (56). Similarly, the coping mechanism can mediate the effect that stress and anxiety, caused by discrimination, on associated physical symptoms (57). Therefore, the absence of a significant association in our study does not necessarily negate the potential impact of perceived discrimination on physical symptoms included in this study but highlights the need for further investigation into potential mediators and moderators including specific cultural factors and coping mechanisms that might influence the relationship between perceived discrimination and physical symptoms among Syrian refugees in Norway.

The present study revealed a significant association between perceived discrimination and adverse mental health outcomes among Syrian refugees in Norway. Previous research among diverse migrant populations also documented the detrimental effects of discrimination on mental well-being, however at different degrees (13, 45, 58). Research has shown that individuals who perceive discrimination in different domains of their lives, such as employment, education, and healthcare, often experience elevated psychological distress (13, 46). This can sabotage a person’s sense of identity, self-worth, and social support systems and lead to chronic stress, which, in turn, contributes to the development of anxiety and depressive symptoms (13), particularly salient among marginalized populations, including refugees and other immigrants, who may already face multiple stressors associated with their displacement and acculturation processes (45). However, the strength of this association could vary and depend on factors such as a sense of belonging to the host country and cultural integration of migrants (12). This study, for the first time, documented the association of experienced discrimination with poor mental health among Syrian refugees with unique cultural disparities and integration processes in the socio-political climates of Norway. Additionally, long-term discrimination exposure may increase vigilance to the point of hypervigilance, which exacerbates stress-related mental health problems (13) such as PSTD symptoms observed in this study.

Refugees have unique healthcare needs that are shaped by their experiences in their country of origin, their migration journey, their host country’s entry and integration policies, and living and working conditions (59). These experiences can increase the vulnerability of refugees to chronic and infectious diseases, making access to healthcare services critical for their well-being (60). On the other hand, perceived discrimination may discourage individuals from seeking and utilizing available health services, exacerbating their health disparities and limiting their access to necessary care (61, 62). Discrimination in healthcare and the utilization of healthcare services among refugees and migrants in Norway is a critical topic that warrants more attention (63, 64). Despite Norway’s comprehensive welfare state with health care provision for all migrants staying legally in the country and emphasis on culturally sensitive care, there are various barriers that some migrants may encounter when accessing and consuming healthcare services (64). These barriers, including discrimination, can contribute to disparities in healthcare utilization and health outcomes among different migrant groups. However, in this study, there was no significant relationship between perceived discrimination and GP and specialist visits. Although one of the limitations of this study was not asking specifically about discrimination experienced in encountering the health system, the absence of a notable association may suggest that primary care in Norway is equitable and accessible to all residents, irrespective of their migrant status.

Despite the lack of association between perceived discrimination and utilization of specialist and GP services, the significant association between perceived discrimination and increased utilization of psychologist visits in our study highlights the unique impact of discrimination on mental health service use. Discrimination experienced by refugees may contribute to psychological distress and mental health concerns, leading individuals to seek specialized mental health support, such as psychologist visits. This is in line with previous studies showing that perceived discrimination is associated with higher rates of mental health service utilization among marginalized populations (65–67). However, in contrast to our finding, a cross-sectional survey among Syrian refugees in Turkey found no significant association between perceived discrimination and mental health service use, despite the high prevalence of mental health symptoms and the gap in treatment was mainly attributed to factors beyond perceived discrimination such as structural (e.g., limited knowledge and difficulties in navigation), linguistic, and cultural obstacles (68). Nonetheless, the complex interplay between perceived discrimination and healthcare-seeking behaviors within the migrant populations in Norway underscores the need for a more thorough investigation. Addtionally, further studies with larger sample size are recommended to specify the use of mental health services and characterize the underlying obstacles, if any, among migrants in need living in Norway.

In summary, a multi-faceted approach is necessary to address discrimination as a public health concern and implement interventions to reduce its prevalence and mitigate its negative consequences among migrant and refugee populations. Efforts should focus on promoting inclusivity, cultural sensitivity, and equal opportunities for all individuals, regardless of their backgrounds (29). Systemic and structural forms of discrimination should be removed by scrutinizing and reforming policies, laws, and institutions, ensuring equal access to resources and opportunities across all sectors, including education, employment, and housing (69). Education and awareness campaigns targeting both the general population, the migrants, and healthcare providers can help foster understanding and combat discriminatory attitudes and behaviors and foster cultural competency among health staff and health literacy among migrants themselves (70).

## Limitations

Although this study contributes to the existing literature by providing a better understanding of the potential impact that discrimination could have on Syrian refugees’ health and their utilization of healthcare in Norway, it is important to acknowledge that the study’s findings should not be interpreted as definitive, as there may still be other sources of bias or unmeasured confounding variables that could affect the results. The cross-sectional design of the study did not allow for any causal inferences. Given the self-reported measures of discrimination, there might be social desirability bias or recall bias and may understimate or overestimate the prevalence of perceived discrimantion and distort its association with study outcomes. Furthermore, it should be noted that the small sample size in the study resulted in low statistical power, which means that the study might not have been able to detect small yet meaningful effects accurately. The high attrition rate might cause a selection bias; for example, if Syrian refugees with lower self-rated health or lower access to the health system had refused to participate in H4I. It is worth noting, however, that such challenges are not uncommon in longitudinal research (71) as the I4H project, even though cross-sectional in design, was the longitudinal follow-up of the CHART project collecting data on the same cohort of Syrian refugees after 4 years settling in Norway. Thus, there is a possibility that there are underlying effects related to perceived discrimination that were not identified due to the limited sample size. However, applying outcome-specific sampling weights in analyses could compensate for such bias to some extent and enhace the validity of our findings. Additionally, it is important to consider that certain factors, such as personality traits, skills, and coping mechanisms, were not assessed in the study. For instance, the study did not investigate how personality traits like resilience or openness might influence individuals’ perceptions of discrimination. Examining these factors could provide a more comprehensive understanding of the complex dynamics involved in perceived discrimination among the studied population. In addition, the frequency of physical symptoms was not explored in this study which may overestimate the rate of physical symptoms if the chronicity is overlooked.

## Conclusion

Syrian refugees in Norway report high rates of discrimination, and those who have experienced discrimination are more likely to suffer from mental health symptoms and have a higher frequency of visits to psychologists. The results can contribute to increasing awareness among policy makers and practitioners and development of targeted interventions and policies aimed at improving the well-being, particulary mental health, and the appropriate integration of Syrian refugees in Norway. Future policies and interventions should aim at promoting social inclusion and reducing discrimination through the implementation of anti-discrimination policies in employment, housing, and education. Additionally, it is important to address the root causes of discrimination, such as negative attitudes towards refugees, stereotyping, and lack of knowledge about refugee issues. Public awareness campaigns and education programs could help to combat these negative attitudes and promote greater understanding and acceptance of refugees.

## Data availability statement

The datasets presented in this article are not readily available because all data and material produced in the present study are stored on the UiB SAFE server and considwred Uib Property and could only be available through a reasonable request upon the approval of the REK and UiB adminstrive entities. Requests to access the datasets should be directed to esperanza.diaz@uib.no.

## Ethics statement

The studies involving humans were approved by the regional committees for medical and health research ethics (REK), Oslo, Norway. The studies were conducted in accordance with the local legislation and institutional requirements. The participants provided their written informed consent to participate in this study.

## Author contributions

OD: Data curation, Formal analysis, Investigation, Methodology, Software, Validation, Visualization, Writing – original draft, Writing – review & editing. ED: Conceptualization, Funding acquisition, Methodology, Project administration, Supervision, Validation, Writing – review & editing, Investigation.
